# Mental Health and Substance Use Among Adults with Disabilities During the COVID-19 Pandemic — United States, February–March 2021

**DOI:** 10.15585/mmwr.mm7034a3

**Published:** 2021-08-27

**Authors:** Mark É. Czeisler, Amy Board, JoAnn M. Thierry, Charles A. Czeisler, Shantha M.W. Rajaratnam, Mark E. Howard, Kristie E.N. Clarke

**Affiliations:** ^1^Turner Institute for Brain and Mental Health and School of Psychological Sciences, Monash University, Melbourne, Australia; ^2^Austin Health, Melbourne, Australia; ^3^Brigham and Women’s Hospital, Boston, Massachusetts; ^4^Harvard Medical School, Boston, Massachusetts; ^5^CDC COVID-19 Response Team; ^6^National Center for Injury Prevention and Control, CDC; ^7^Epidemic Intelligence Service, CDC; ^8^University of Melbourne, Melbourne, Australia.

Adults with disabilities, a group including >25% of U.S. adults (*1*), experience higher levels of mental health and substance use conditions and lower treatment rates than do adults without disabilities* (*2*,*3*). Survey data collected during April–September 2020 revealed elevated adverse mental health symptoms among adults with disabilities (*4**)* compared with the general adult population (*5*). Despite disproportionate risk for infection with SARS-CoV-2, the virus that causes COVID-19, and COVID-19–associated hospitalization and mortality among some adults with disabilities (*6*), information about mental health and substance use in this population during the pandemic is limited. To identify factors associated with adverse mental health symptoms and substance use among adults with disabilities, the COVID-19 Outbreak Public Evaluation (COPE) Initiative^†^ administered nonprobability–based Internet surveys to 5,256 U.S. adults during February–March 2021 (response rate = 62.1%). Among 5,119 respondents who completed a two-item disability screener, nearly one third (1,648; 32.2%) screened as adults with disabilities. These adults more frequently experienced symptoms of anxiety or depression (56.6% versus 28.7%, respectively), new or increased substance use (38.8% versus 17.5%), and suicidal ideation (30.6% versus 8.3%) than did adults without disabilities. Among all adults who had received a diagnosis of mental health or substance use conditions, adults with disabilities more frequently (42.6% versus 35.3%; p <0.001) reported that the pandemic made it harder for them to access related care or medication. Enhanced mental health and substance use screening among adults with disabilities and improved access to medical services are critical during public health emergencies such as the COVID-19 pandemic.

During February 16–March 8, 2021, among 8,475 eligible invited respondents aged ≥18 years, 5,261 (62.1%) completed nonprobability based, English-language, Internet-based Qualtrics surveys for COPE.^§^ Participants provided informed consent electronically. Quota sampling and survey weighting were used to match U.S. Census Bureau’s 2019 American Community Survey adult U.S. population estimates for sex, age, and race/ethnicity to enhance the representativeness of this nonrandom sample. 

Among 5,256 respondents who answered questions for weighting variables, 5,119 (97.4%) completed a two-question disability screener.^¶^ Respondents completed clinically validated self-screening instruments for symptoms of anxiety and depression** and reported past-month new or increased substance use to cope with stress or emotions and serious suicidal ideation.^††^ Respondents also indicated prepandemic and past-month use of seven classes^§§^ of substances to cope with stress or emotions. Adults with diagnosed anxiety, depression, posttraumatic stress disorder, or substance use disorders indicated whether their ability to access care or medications for these conditions was easier, harder, or unaffected because of the pandemic. Prevalence estimates for adverse mental health symptoms and substance use were compared among adults with and without disabilities using chi-square tests. Multivariable Poisson regression models with robust standard error estimators were used to estimate adjusted prevalence ratios (aPRs) by symptom type among adults with and without disabilities. To calculate associations between disability status and adverse mental health symptoms or substance use over time, aPRs were estimated for symptoms among unique participants in previous COPE survey waves (June, September, and December 2020). Covariates^¶¶^ included sex, age group, race/ethnicity, income, U.S. Census region, urbanicity, and parental or unpaid caregiving roles.*** McNemar’s test assessed prepandemic and past-month substance use among adults with and without disabilities. Analyses were conducted using Python software (version 3.7.8; Python Software Foundation) and R statistical software (version 4.0.2; R Foundation) using the R survey package (version 3.29; R Foundation). The Monash University Human Research Ethics Committee reviewed and approved the study. This activity was reviewed by CDC and conducted consistent with applicable federal law and CDC policy.^†††^

Among a total of 5,119 respondents, 1,648 (32.2%) respondents reported living with disabilities (778 [47.2%] with limiting physical, mental, or emotional conditions only; 171 [10.4%] with health conditions requiring special equipment only; and 669 [42.4%] with both types of conditions) (Table). Overall, 64.1% of adults with disabilities reported adverse mental health symptoms or substance use compared with 36.0% of adults without disabilities; past-month substance use was higher among adults with disabilities (40.6%) than among adults without disabilities (24.5%). Prevalence estimates of each of the following were higher among adults with disabilities than among adults without disabilities: symptoms of anxiety or depression (56.6% versus 28.7%, respectively), new or increased substance use (38.8% versus 17.5%), and serious suicidal ideation (30.6% versus 8.3%) (Supplementary Table, https://stacks.cdc.gov/view/cdc/108999). At all timepoints, aPRs for all symptom types were significantly higher among adults with disabilities than among adults without disabilities (Figure 1). During February 16–March 8, 2021, among adults with disabilities, aPRs for symptoms of anxiety or depression and new or increased substance use were approximately 1.5 times as high, and the aPR for serious suicidal ideation was approximately 2.5 times as high as in adults without disabilities. Comparing subgroups of adults with and without disabilities, symptoms of anxiety or depression were approximately twice as prevalent among adults with disabilities who were aged ≥50 years (aPR = 2.4; 95% confidence interval [CI] = 1.7–3.2), those of non-Hispanic Asian race/ethnicity (2.4; 95% CI = 1.3–4.8), those of Hispanic or Latino (Hispanic) ethnicity (2.1; 95% CI = 1.4–3.0), and those who were not in parental or caregiver roles (2.1; 95% CI = 1.7–2.6). New or increased substance use was approximately twice as prevalent among adults with disabilities in parental roles only (2.4; 95% CI = 1.5–3.9) and among essential workers (2.3; 95% CI = 2.0–2.7). Suicidal ideation was also more prevalent among adults with disabilities aged ≥50 years (4.0; 95% CI = 2.1–7.8), those of Hispanic ethnicity (3.4; 95% CI = 1.9–6.0), adults in unpaid caregiving roles (3.4; 95% CI = 1.5–7.7), and essential (3.5; 95% CI = 2.8–4.4) or nonessential (5.3; 95% CI = 2.8–10.1) workers.

**TABLE:** Prevalence of symptoms of anxiety or depression, substance use, and suicidal ideation among adults with disabilities, by disability status and other characteristics — United States, February 16–March 8, 2021

Characteristic	No. (%)		Adults with disabilities, No. (%)*			
	All respondents	Adults with disabilities	Symptoms of anxiety or depression^†^	New or increased substance use to cope^§^	Seriously considered suicide^¶^	One or more of these symptoms
**Total**	**5,119 (100)**	**1,648 (32.2)**	**932 (56.6)**	**640 (38.8)**	**504 (30.6)**	**1,057 (64.1)**
Disability screener**						
Limited by a physical, mental, or emotional condition	778 (15.2)	778 (47.2)	417 (53.7)	218 (28.0)	148 (19.0)	465 (59.8)
Limited by a health condition that requires special equipment	171 (3.3)	171 (10.4)	104 (60.5)	88 (51.5)	65 (38.2)	123 (71.8)
Both of above	699 (13.7)	669 (42.4)	411 (58.8)	334 (47.8)	291 (41.5)	469 (67.1)
Neither of above	3,471 (67.8)	0 (—)	N/A	N/A	N/A	N/A
**Sex** ^††^						
Female	2,499 (48.8)	789 (47.9)	445 (56.5)	260 (32.9)	178 (22.6)	501 (63.5)
Male	2,583 (50.5)	838 (50.8)	469 (55.9)	369 (44.0)	314 (37.4)	537 (64.1)
**Age group**, **yrs**						
18–29	938 (18.3)	314 (19.0)	250 (79.8)	185 (59.1)	136 (43.3)	276 (87.8)
30–39	967 (18.9)	325 (19.7)	259 (79.8)	198 (60.9)	166 (51.1)	281 (86.6)
40–49	818 (16.0)	253 (15.4)	180 (70.9)	137 (54.0)	125 (49.5)	202 (79.6)
50–59	972 (19.0)	309 (18.8)	132 (42.6)	80 (25.9)	54 (17.5)	158 (51.2)
60–69	790 (15.4)	235 (14.2)	59 (25.2)	21 (8.9)	4 (1.8)	72 (30.7)
≥70	634 (12.4)	213 (12.9)	52 (24.7)	19 (8.8)	19 (8.8)	68 (31.9)
**Race/Ethnicity**						
White, non-Hispanic	3,103 (60.6)	975 (59.2)	522 (53.6)	327 (33.5)	266 (27.3)	585 (60.0)
Black, non-Hispanic	638 (12.5)	181 (11.0)	99 (54.6)	68 (37.9)	35 (19.3)	110 (60.9)
Asian, non-Hispanic	289 (5.6)	65 (3.9)	39 (61.1)	18 (27.8)	14 (21.0)	47 (72.1)
Multiple/other race, non-Hispanic^§§^	188 (3.7)	70 (4.3)	32 (45.2)	16 (23.3)	13 (18.3)	32 (45.8)
Hispanic or Latino, any race	902 (17.6)	357 (21.7)	240 (67.2)	210 (58.8)	177 (49.5)	283 (79.3)
**2020 Household income, USD** ^¶¶^						
<25,000	1,182 (23.1)	544 (33.0)	286 (52.6)	151 (27.8)	107 (19.7)	327 (60.0)
25,000–49,999	1,203 (23.5)	355 (21.5)	179 (50.4)	110 (30.9)	82 (23.2)	202 (56.9)
50,000–99,999	1,306 (25.5)	350 (21.2)	191 (54.6)	134 (38.2)	103 (29.5)	218 (62.1)
≥100,000	1,204 (23.5)	341 (20.7)	253 (74.1)	232 (68.1)	205 (60.1)	286 (83.8)
**Education**						
High school diploma or less	1,379 (26.9)	485 (29.4)	264 (54.4)	155 (31.8)	135 (27.9)	309 (63.7)
College or some college	2,876 (56.2)	865 (52.5)	463 (53.5)	312 (36.0)	213 (24.6)	520 (60.1)
After bachelor's degree	865 (16.9)	298 (18.1)	206 (69.0)	174 (58.2)	156 (52.3)	228 (76.4)
**Employment status**						
Employed (essential employee)	1,797 (35.1)	605 (36.7)	475 (78.6)	448 (74.2)	371 (61.4)	542 (89.6)
Employed (nonessential employee)	941 (18.4)	151 (9.1)	87 (57.9)	53 (35.2)	38 (25.4)	103 (68.3)
Unemployed	936 (18.3)	349 (21.2)	190 (54.5)	77 (22.2)	55 (15.9)	207 (59.3)
Retired	1,263 (24.7)	493 (29.9)	142 (28.8)	45 (9.1)	24 (4.8)	167 (33.8)
Student	182 (3.6)	51 (3.1)	38 (73.7)	16 (31.9)	15 (29.8)	38 (74.5)
Parental role and unpaid caregiving status***						
Neither parent nor caregiver	2,882 (56.3)	741 (44.9)	294 (39.7)	90 (12.2)	70 (9.4)	323 (43.6)
Parent only	611 (11.9)	189 (11.5)	97 (51.3)	48 (25.1)	21 (11.3)	110 (58.0)
Caregiver role of adults only	426 (8.3)	117 (7.1)	57 (48.6)	39 (33.1)	24 (20.9)	71 (60.5)
Parental and caregiver roles	1,201 (23.5)	602 (36.5)	485 (80.5)	463 (77.0)	389 (64.6)	553 (92.0)
**U.S. Census region** ^†††^						
Northeast	899 (17.6)	267 (16.2)	177 (66.0)	119 (44.7)	109 (40.6)	188 (70.5)
Midwest	1,069 (20.9)	349 (21.1)	208 (59.8)	126 (36.0)	94 (27.1)	222 (63.6)
South	2,074 (40.5)	700 (42.5)	367 (52.4)	262 (37.4)	195 (27.9)	442 (63.1)
West	1,077 (21.0)	333 (20.2)	180 (54.2)	133 (40.1)	106 (31.8)	205 (61.7)
**Urbanicity** (n = 5,091)^§§§^						
Urban	4,241 (83.3)	1,313 (79.6)	761 (58.0)	544 (41.4)	440 (33.5)	866 (66.0)
Rural	850 (16.7)	322 (19.5)	158 (49.1)	87 (27.1)	56 (17.4)	178 (55.2)

**Abbreviations**: N/A = not applicable; USD = U.S. dollars.

* Weighted rounded counts and percentages might not sum to expected values.

^†^ Symptoms of anxiety and depression were assessed via the four-item Patient Health Questionnaire (PHQ-4). Respondents who scored ≥3 out of 6 on the Generalized Anxiety Disorder (GAD-2) and Patient Health Questionnaire (PHQ-2) subscales were considered symptomatic for these respective conditions.

^§^ New or increased substance use was assessed by using the question, “Have you started or increased using substances to help you cope with stress or emotions during the COVID-19 pandemic? Substance use includes alcohol, legal or illegal drugs, or prescription drug use in any way not directed by a doctor.”

^¶^ Suicidal ideation was assessed by using an item from the National Survey on Drug Use and Health (https://nsduhweb.rti.org/respweb/homepage.cfm) adapted to refer to the previous 30 days, “At any time in the past 30 days, did you seriously think about trying to kill yourself?”

** Adults who had a disability were defined as such based on a qualifying response to either one of two questions: “Are you limited in any way in any activities because of physical, mental, or emotional condition?” and “Do you have any health conditions that require you to use special equipment, such as a cane, wheelchair, special bed, or special telephone?” Respondents who completed only one of the two disability screening questions (limited by a physical, mental, or emotional condition: 17); limited by a health condition that requires special equipment: 12) were classified as living with only that disability. https://www.cdc.gov/brfss/questionnaires/pdf-ques/2015-brfss-questionnaire-12-29-14.pdf

^††^ Gender responses of “Transgender” (22; 0.4%) and “None of these” (15; 0.3%) are not shown because of small counts.

^§§^ The non-Hispanic, multiple/other race or multiple races category includes respondents who identified as not Hispanic and as more than one race or as American Indian or Alaska Native, Native Hawaiian or Other Pacific Islander, or any other race.

^¶¶^ Household income responses of “Prefer not to say” (225) are not shown because of an inability to sufficiently characterize these responses.

*** Adults who were in parental or unpaid caregiving roles were self-identified. For this analysis, the definition of unpaid caregivers of adults was having provided unpaid care to a relative or friend ≥18 years to help them take care of themselves at any time during the 3 months before the survey. The definition of someone in a parental role was having provided unpaid care to a relative or friend <18 years. Respondents answered these questions separately. During analysis, all respondents were categorized as being in a parental role only, caregivers of adults only, having both parental and caregiving roles, or having neither parental nor caregiving roles. Adults in parenting roles might not have been natural or legal parents of children in their care.

^†††^ https://www2.census.gov/geo/pdfs/maps-data/maps/reference/us_regdiv.pdf

^§§§^ Invalid postcodes were provided by 28 respondents, for whom urbanicity was not categorized. https://www.hrsa.gov/rural-health/about-us/definition/datafiles.html

**FIGURE 1:**
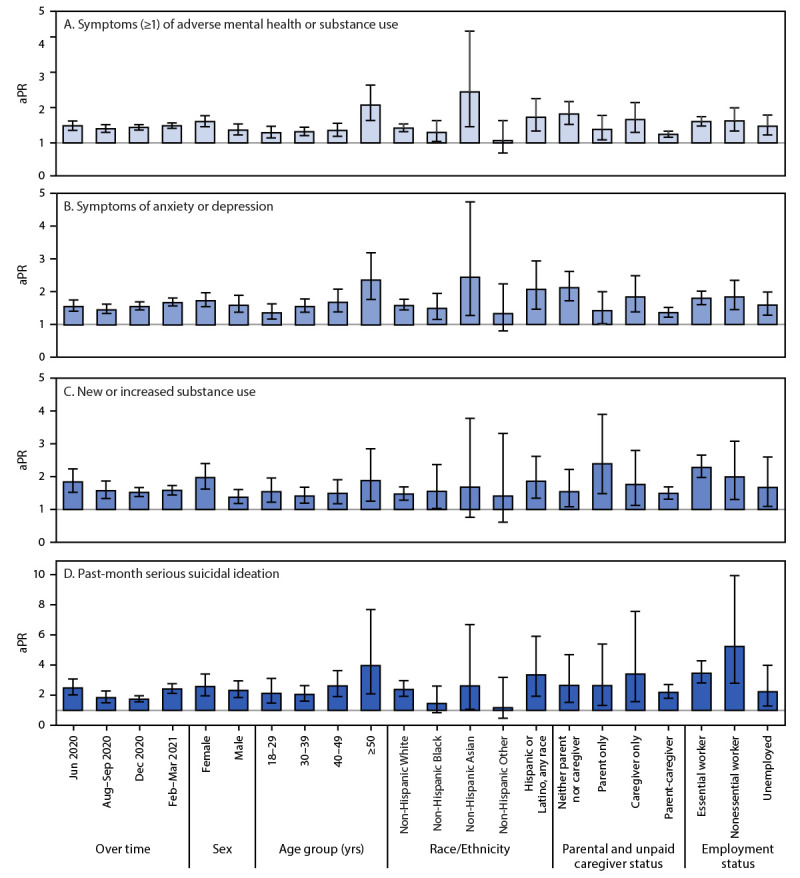
Adjusted prevalence ratios* and 95% confidence intervals^†^ for ≥1 symptoms of adverse mental health or substance use (A), symptoms of anxiety or depression (B), new or increased substance use (C), and suicidal ideation (D) among adults with disabilities, compared with adults without disabilities (referent group)^§^ — United States, February 16–March 8, 2021^¶^ **Abbreviations**: aPR = adjusted prevalence ratio; CI = confidence interval. * With 95% CIs indicated by error bars. Multivariable Poisson regression models included sex, age group in years, race/ethnicity, income, U.S. Census region, urbanicity, and parental or unpaid caregiving roles (parental roles were not assessed in June 2020; only unpaid caregiving roles were considered for this variable in the June 2020 models). Separate, additional models were run to estimate aPRs for the following employment statuses: essential worker, nonessential worker, and unemployed. Estimates were not made for retired or student employment statuses because of collinearity between these employment statuses and age. ^†^ For panels A, B, and C, the y-axis range for aPR estimates is 0–5, which contains all aPRs and 95% CIs for these panels with maximal view of differences in model estimates. For panel D, given the relative rarity of suicidal ideation among some demographic subgroups that results in wide CIs for aPR estimates, the y-axis range is 0–10. ^§^ Within each subgroup, adults without disabilities are the reference group used to estimate aPRs for outcomes among adults with disabilities. ^¶^ Estimated aPRs are during February 16–March 8, 2021, except for the “over time” estimates, which also include estimates based on data collected during June 24–30, 2020, August 28–September 6, 2020, and December 6–27, 2020.

The prevalence of substance use to cope with stress or emotions among adults with disabilities was higher than that among adults without disabilities, both prepandemic (39.7% versus 25.3%, respectively) and in the past month (40.6% versus 24.5%; both p<0.001) (Figure 2). Among adults with disabilities, the past-month prevalence of methamphetamine use (8.4%), nonopioid prescription drug misuse (4.9%), and polysubstance use (16.9%) was approximately twice as high, and the prevalence of cocaine use (6.4%) and prescription or illicit opioid use (9.1%) were nearly three times as high compared with those among adults without disabilities (methamphetamine use 3.4%; nonopioid prescription drug misuse 2.0%; polysubstance use 7.9%; cocaine use 2.2%; prescription or illicit opioid use 3.2%). Past-month methamphetamine use prevalence increased significantly compared with prepandemic use prevalence among all respondents (with disabilities, 45.6% increase, p<0.001; without disabilities, 40.6% increase, p = 0.003). Among respondents who reported a diagnosed mental health or substance use condition, a higher percentage of adults with (versus without) disabilities reported that accessing care or medication was harder because of the COVID-19 pandemic (42.6% versus 35.3%, respectively, p<0.001).

**FIGURE 2:**
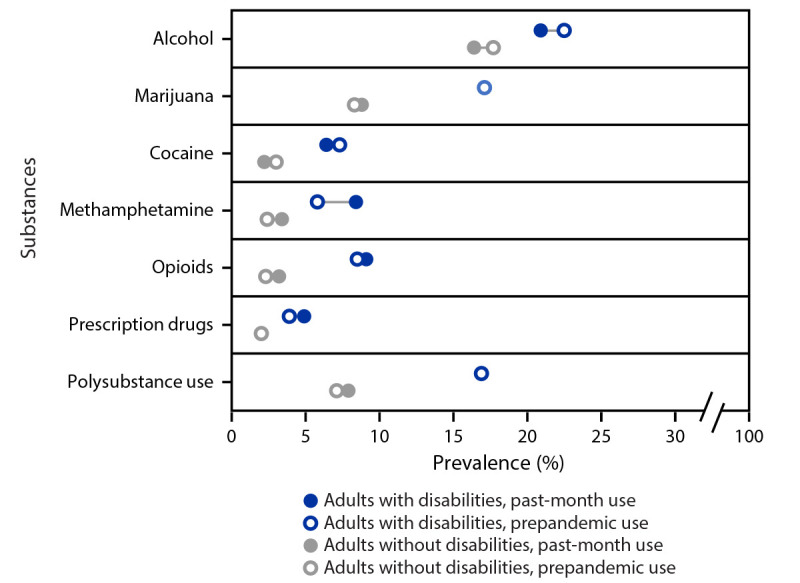
Prevalence of prepandemic and past-month substance use to cope with stress or emotions among adults, by disability status and type of substance — United States, February 16–March 8, 2021*^,^^†^^,^^§^ * Overall, prepandemic and past-month use of any of these substances were reported by 39.7% and 40.6%, respectively, of adults with disabilities, and by 25.3% and 24.5%, respectively, of adults without disabilities. ^†^ All differences between adults with disabilities and adults without disabilities were significant (chi-square p-value <0.05). ^§^ Circles for use of marijuana (among adults with disabilities), use of prescription drugs (among adults without disabilities), and polysubstance use (among adults with disabilities) might appear overlapping because of very small changes in reported prevalence (<1% in all cases).

## Discussion

Nearly two thirds of surveyed adults with disabilities (who represented approximately 32% of the sample) reported adverse mental health symptoms or substance use in early 2021, compared with approximately one third of adults without disabilities. Serious suicidal ideation was approximately 2.5 times as high among adults with disabilities, and methamphetamine use, opioid use, nonopioid prescription drug misuse, and polysubstance use were at least twice as prevalent among adults with disabilities. These findings suggest value in enhanced mental health screening among adults with disabilities and in ensuring accessibility of routine and crisis services, particularly given that many adults reported that the COVID-19 pandemic had reduced mental health and substance use care or medication accessibility. Mental health disparities among adults with disabilities were observed across demographic groups, highlighting the importance of ensuring access to disaster distress^§§§^ and suicide prevention^¶¶¶^ resources in this population. Important strategies to prevent persons from becoming suicidal include strengthening economic supports, promoting connectedness, and teaching coping skills.**** Health care providers could incorporate trauma-informed care, because adults with disabilities might have encountered stigma and trauma in previous health care interactions. Adults with disabilities more frequently reported prepandemic and past-month substance use to cope with stress or emotions compared with adults without disabilities. The substance with the largest increase in use was methamphetamine, which is particularly concerning given the increase in amphetamine overdoses^††††^ (*7*). Drug overdose deaths rose in 2020, driven by synthetic opioids.^§§§§^ Consistent with previous research, adults with disabilities disproportionately reported opioid use and nonopioid prescription drug misuse (*8*), highlighting the importance of educating patients and ensuring clinician access to prescription drug monitoring programs.^¶¶¶¶^ Nearly one in ten adults with disabilities reported past-month opioid use, and opioid use among adults without disabilities increased. Policies that reduce barriers to evidence-based treatment, including recently updated buprenorphine practice guidelines,***** might improve access.

The findings in this report are subject to at least four limitations. First, self-reported mental health and substance use might be subject to social desirability biases and stigma, which could lead to underreporting. Second, because the surveys were English-language only and data were obtained using nonprobability–based sampling, despite quota sampling and survey weighting, the findings from this nonrandom sample might not be generalizable. However, the proportion and demographics of surveyed adults with disabilities were similar to those of recent samples from other sources with the same or similar screening questions (*1*,*2*,*4*), and prevalence estimates of symptoms of anxiety and depression were largely consistent with those from other sources for the U.S. adult population (*9*) and adults with disabilities (*4*) including the U.S. Census Bureau’s probability-based Household Pulse Survey (64.3% among adults with disabilities compared with 27.4% among adults without disabilities in April 2021).^†††††^ Third, the respondents with disabilities might not be representative of all adults with disabilities, some of whom might lack access to hardware or assistive technologies required to independently complete the survey. Finally, adverse mental health symptoms might, in some cases, represent respondents’ disabling mental health conditions, which could confound associations with other comorbid disabling conditions (e.g., physical, cognitive, sensory); however, sensitivity analyses excluding adults with disabilities who had mental health or substance use diagnoses yielded consistent findings.

Adults with disabilities have been disproportionately affected by adverse mental health symptoms and substance use during the COVID-19 pandemic, highlighting the importance of improved access to treatment for this population. Clinicians might consider screening all patients for mental health and substance use conditions during and after the pandemic.^§§§§§^ Behavioral health care providers might also consider facility, policy, and procedural pathway analyses to ensure accessibility for clients with physical, sensory, or cognitive disabilities.^¶¶¶¶¶^ Strategies designed to increase access to care and medication during public health emergencies, such as telehealth, might consider telemedicine platform and system accessibility for adults with disabilities (*10*); further research to identify and address health disparities among adults with disabilities could help guide additional evidence-based strategies.

**Summary**

**What is already known about this topic?**

Adults with disabilities experience higher levels of mental health conditions and substance use than do adults without disabilities.

**What is added by this report?**

During February–March 2021, 64.1% of surveyed U.S. adults with disabilities reported adverse mental health symptoms or substance use; past-month substance use was higher than that among adults without disabilities (40.6% versus 24.5%, respectively). Among adults with a diagnosis of mental health or substance use conditions, adults with disabilities more frequently (43% versus 35%) reported pandemic-related difficulty accessing related care and medications.

**What are the implications for public health practice?**

During public health emergencies, including the COVID-19 pandemic, enhanced mental health and substance use screening among adults with disabilities and improved access to related health care services are critical.

## Footnotes

*
https://store.samhsa.gov/sites/default/files/d7/priv/pep19-02-00-002_508_022620.pdf

† The COVID-19 Outbreak Public Evaluation (COPE) Initiative (https://www.thecopeinitiative.org/) is designed to assess public attitudes, behaviors, and beliefs related to COVID-19 pandemic and to evaluate mental and behavioral health during the pandemic. The COPE Initiative surveys included in this analysis were administered by Qualtrics, LLC (https://www.qualtrics.com), a commercial survey company with a network of participant pools with varying recruitment methodologies that include digital advertisements and promotions, word-of-mouth and membership referrals, social networks, television and radio advertisements, and offline mail-based approaches.

§ Eligibility to complete surveys was determined after electronic contact of potential participants with inclusion criteria of age ≥18 years and residence within the United States.

¶ Disability was defined as such based on a qualifying response by an adult to either one of two questions: “Are you limited in any way in any activities because of physical, mental, or emotional condition?” and “Do you have any health conditions that require you to use special equipment, such as a cane, wheelchair, special bed, or special telephone?” https://www.cdc.gov/brfss/questionnaires/pdf-ques/2015-brfss-questionnaire-12-29-14.pdf

** Symptoms of anxiety and depression were assessed with the four-item Patient Health Questionnaire (PHQ-4). Respondents who scored ≥3 out of 6 on the Generalized Anxiety Disorder (GAD-2) and Patient Health Questionnaire (PHQ-2) subscales were considered symptomatic for the respective conditions.

†† New or increased substance use was assessed with the question, “Have you started or increased using substances to help you cope with stress or emotions during the COVID-19 pandemic? Substance use includes alcohol, legal or illegal drugs, or prescription drug use in any way not directed by a doctor.” Suicidal ideation was assessed with an item from the National Survey on Drug Use and Health (https://nsduhweb.rti.org/respweb/homuepage.cfm) adapted to refer to the preceding 30 days, “At any time in the past 30 days, did you seriously think about trying to kill yourself?”

§§ Alcohol, marijuana, cocaine, methamphetamine, prescription or illicit opioids, benzodiazepines, and prescription drugs other than opioids used in a way not directed by a doctor.

¶¶ Models to estimate aPRs for adverse mental health symptoms and substance use were run with each of the collinear variables income and education during preliminary analysis. Estimated aPRs did not differ meaningfully. In the report, the models including income were included to account for potential differences in access to health care more directly. To avoid collinearity with age, employment status was included in a separate model, and aPRs were not estimated for retired status or student employment status.

*** Adults who were in parental or unpaid caregiving roles were self-identified. For this analysis, the definition of unpaid caregivers of adults was having provided unpaid care to a relative or friend aged ≥18 years to help them take care of themselves at any time during the three months before the survey. The definition of someone in a parental role was having provided unpaid care to a relative or friend aged <18 years. Respondents were categorized as being in a parental role only, a caregiver of adults role only, having both parental and caregiving roles, or having neither parental nor caregiving roles. Adults in parenting roles might not have been biologic or adoptive parents of the children.

††† 45 C.F.R. part 46, 21 C.F.R. part 56; 42 U.S.C. Sect. 241(d); 5 U.S.C. Sect. 552a; 44 U.S.C. Sect. 3501 et seq.

§§§ Substance Abuse and Mental Health Services Administration National Helpline (https://www.samhsa.gov/find-help/national-helpline); Disaster Distress Helpline (https://www.samhsa.gov/disaster-preparedness).

¶¶¶ National Suicide Prevention Lifeline: 1–800–273-TALK for English, 1–888–628–9454 for Spanish, or Lifeline Crisis Chat (https://suicidepreventionlifeline.org/chat/).

****
https://www.cdc.gov/violenceprevention/pdf/suicideTechnicalPackage.pdf

††††
https://emergency.cdc.gov/han/2020/han00438.asp

§§§§
https://www.cdc.gov/media/releases/2020/p1218-overdose-deaths-covid-19.html

¶¶¶¶
https://www.cdc.gov/drugoverdose/pdmp/states.html

*****
https://www.federalregister.gov/documents/2021/04/28/2021-08961/practice-guidelines-for-the-administration-of-buprenorphine-for-treating-opioid-use-disorder

†††††
https://www.cdc.gov/nchs/covid19/pulse/functioning-and-disability.htm

§§§§§ https://www.uspreventiveservicestaskforce.org/uspstf/recommendation/drug-use-illicit-screening; https://www.uspreventiveservicestaskforce.org/uspstf/recommendation/depression-in-adults-screening
